# Glow Discharge Plasma Treatment on Zirconia Surface to Enhance Osteoblastic-Like Cell Differentiation and Antimicrobial Effects

**DOI:** 10.3390/ma13173771

**Published:** 2020-08-26

**Authors:** Yu-Hwa Pan, Jerry Chin Yi Lin, Mei Kuang Chen, Eisner Salamanca, Cheuk Sing Choy, Pei-Yo Tsai, Sy-Jye Leu, Kai-Chiang Yang, Haw-Ming Huang, Wan-Ling Yao, Wei Jen Chang

**Affiliations:** 1 School of Dentistry, College of Oral Medicine, Taipei Medical University, Taipei 110, Taiwan; shalom.dc@msa.hinet.net (Y.-H.P.); drjerrylin@tmu.edu.tw (J.C.Y.L.); m204107008@tmu.edu.tw (M.K.C.); eisnergab@tmu.edu.tw (E.S.); m204102011@tmu.edu.tw (P.-Y.T.); hhm@tmu.edu.tw (H.-M.H.); ywl0616@tmu.edu.tw (W.-L.Y.); 2 Department of General Dentistry, Chang Gung Memorial Hospital, Taipei 106, Taiwan; 3 Graduate Institute of Dental & Craniofacial Science, Chang Gung University, Taoyuan 333, Taiwan; 4 School of Dentistry, College of Medicine, China Medical University, Taichung 404, Taiwan; 5 Department of Community Medicine, En Chu Kong Hospital, New Taipei City 237, Taiwan; 6 Department of Nursing, Yuanpei University of Medical technology, Hsin Chu, Taipei 300, Taiwan; 7 Department of Microbiology and Immunology, School of Medicine, College of Medicine, Taipei Medical University, Taipei 110, Taiwan; cmbsycl@tmu.edu.tw; 8 School of Dental Technology, College of Oral Medicine, Taipei Medical University, Taipei 110, Taiwan; pumpkin@tmu.edu.tw; 9 Dental Department, Taipei Medical University, Shuang-Ho hospital, Taipei 235, Taiwan

**Keywords:** zirconia, glow discharge plasma, osteoblastic-like cell differentiation, peri-implantitis

## Abstract

Peri-implantitis is the pathological condition of connective tissue inflammation and the progressive loss of supporting bone around dental implants. One of the primary causes of peri mucositis evolving into peri-implantitis is bacterial infection, including infection from *Porphyromonas gingivalis*. Enhancing the surface smoothness of implants helps to prevent *P. gingivalis* adhesion to the implant’s surface. Interaction analyses between bacteria and the surface roughness of zirconia (Zr) discs subjected to a glow discharge plasma (GDP) treatment compared with non-plasma-treated autoclaved control Zr discs were done. Examinations of the material prosperities revealed that the GDP-treated Zr group had a smoother surface for a better wettability. The GDP-treated Zr discs improved the proliferation of the osteoblast-like cells MG-63, and the osteoblastic differentiation was assessed through alkaline phosphatase detection and marker gene bone sialoprotein (Bsp) and osteocalcin (OC) induction. Scanning electron microscopy demonstrated a relatively low *P. gingivalis* adhesion on GDP-treated Zr disks, as well as lower colonization of *P. gingivalis* compared with the control. Our findings confirmed that the GDP treatment of Zr discs resulted in a significant reduction of *P. gingivalis* adhesion and growth, demonstrating a positive correlation between surface roughness and bacteria adhesion. Therefore, the GDP treatment of Zr dental implants can provide a method for reducing the risk of peri-implantitis.

## 1. Introduction

Despite improvements in dental care, millions of people worldwide suffer tooth loss, predominantly due to tooth decay, periodontal disease, or injury. The availability of dental implants represents the support for functional prosthesis and patients’ satisfaction with esthetic appearance [1,2,3,4]. However, after implantation, biological and physical complications may harm or induce damage to oral tissue [5,6,7,8]. Bacterial infection is the primary cause of complications involving oral inflammation [9,10,11,12,13]. This infection may be induced by opportunistic and common periodontopathogenic bacteria proliferation on the surface of the implant, developing the plaque-associated pathological condition known as peri-implantitis [14]. Clinically, peri-implantitis can be triggered by bacteria and can be detected by finding bleeding while probing, together with bone loss on radiographs around dental implants [15,16,17,18,19]. Spirochetes have been reported to cause peri-implantitis in the same manner as Gram-negative anaerobe bacteria, especially *Porphyromonas gingivalis* [9,20,21]. *P. gingivalis* is a well-known pathogen involved in periodontitis progression and the invasion of host cells to cause the destructive events involved in the peri-implantitis disease [22]. The progression of oral plaque and microbiota, also called biofilms that accumulate on the implant surface, induces peri-implantitis, resulting in damage to oral tissues [23,24]. The biofilm is a sessile community of cells that produce a matrix of extracellular polymeric substances. The implant surface is roofed by a pellicle layer, which is an organic stratum consisting of lipids, proteins, and glycoproteins that initiate bacterial colonization and biofilm formation. In clinical observations, patients with peri-implantitis demonstrate a high risk of bone destruction around implants. Therefore, it is imperative to prevent bacterial infection and identify treatments to regenerate the soft and hard tissues to restore the implant. The accumulation of microbial infection leads to the failure of dental implants; therefore, studies are needed to investigate effective preventative therapies.

Regarding the identification of appropriate therapies to reduce peri-implantitis, several studies have concentrated on separate aspects of blocking or reducing dental biofilm formation and accumulation [16,17,18]. Some studies have reported that using metal curettes and conventional ultrasonic scalers improves the bone–implant interface conditions by removing plaque and calculus; however, these techniques often damage the implant surface [25,26,27,28,29,30,31]. Other studies have shown air abrasives produce less damage, just like non-metallic instruments, cannot eliminate plaque and calculus [28,32]. Glow discharge plasma (GDP) treatments are often applied for cleaning, preparing, and modifying biomaterials [33]. Some studies have demonstrated that the GDP treatment of Ti implant surfaces increases cell adhesion and spread whilst improving the implant surface roughness, wettability, functional protein configuration, and creating biofunctional groups [34,35,36]. In addition, a GDP treatment can reduce the contact angle, eliminate residues from the implant surface while maintaining its physicochemical properties, such as hydrocarbon content, functional hydroxyl groups, and surface free energy. Zirconia (Zr) is a commonly used material in dental implant fixtures and as an abutment for oral rehabilitation [37,38]. Furthermore, studies have found that GDP-treated Zr implants induced hydrophilicity and enhanced the process of osseointegration [34,39]. Implant surface properties have been shown to play a critical role in microbial infection and influence oral health. In the present study, we examined the interaction between *P. gingivalis* and the surface roughness of zirconia (Zr) discs subjected to a glow discharge plasma (GDP) treatment compared with non-plasma-treated autoclaved Zr discs (used as a control).

## 2. Materials and Methods

### 2.1. Zr Disk Preparation

Zr disks (Coho Technology Co. Ltd., Taipei, Taiwan; diameter: 10 mm and thickness: 1 mm) were immersed in a detergent solution and cleaned using ultrasound and rinsed with distilled water. The disks were submerged in acetone, followed by two washes with distilled water. Finally, the Zr disks were sterilized using an autoclave and dried. The samples were randomly divided into two groups: Control (Ctrl) disks were only sterilized, while the GDP-treated disks were autoclaved and then subjected to a GDP treatment.

### 2.2. GDP Treatment

The disk surfaces were treated in a plasma reactor (AST Products Inc., North Billerica, MA, USA) for the GDP treatment. The Zr disk surfaces were treated via GDP using argon (Ar-GDP) at 85 W and 13.56 MHz under 100 millitorr for 15 min [40].

### 2.3. SEM Surface Analysis

All disks were coated with nanogold particles, placed in a specimen stub, and studied using SEM with an accelerating voltage of 20 kV in vacuum mode and a secondary electron image (SU-3500; Hitachi Ltd., Kyoto, Japan). Five random areas of the disks were chosen at 1000× magnification for the microphotograph samples.

### 2.4. Energy-Dispersive Spectrometry (EDS)

The discs’ surface compositions were examined using an energy-dispersive X-ray spectrometer (EDS; Model 3500; Hitachi, Ltd., Tokyo, Japan).

### 2.5. Electron Spectroscopy for Chemical Analysis (XPS)

The surface composition in the disks was determined via XPS using 1486.6 eV X-rays. The work was done using an oil-free pumped VG Scientific ESCALAB 200X electron spectrometer (VG Scientific, East Grinstead, UK). Spectra were acquired using Al Kα or Mg Kα excitation (15 kV, 20 mA) at a take-off angle of 15° relative to the sample normal. This technique is among the most utilized techniques used for the characterization of solid surfaces [41].

### 2.6. Attenuated Total Reflectance—Fourier Transform Infrared Spectroscopy (ATR-FTIR)

ATR-FTIR was performed using a Nicolet iS5 (Thermo Fisher Scientific, Madison, WI, USA) equipped with an iD7 crystal ZnSe in reflection mode, and the absorbance of spectra was measured using 16 scans coded with a 0.482 cm^−1^ resolution in the wavenumber region 4000–650 cm^−1^.

### 2.7. Examination of Wettability

The wettability of the disks was measured via static water contact angles on the Zr discs of each of the two groups using a 35-mm camera to illuminate the disk. A 4 μL water droplet (Millipore-Q, Millipore, Bedford, MA, USA; filtered, 20 °C) at a body temperature of 37 °C was settled on top of each surface and the contact angle was measured and quantified.

### 2.8. Examination of the Surface Roughness

All surface roughness measurements were performed using a profilometer (TR200, An-Bomb instrument CO., Ltd., Tainan, Taiwan). The program was set according to the manufacturer’s protocol and was used to measure the surface roughness (Ra) of the two groups of Zr disc surfaces. One measurement was conducted for each specimen in the *X* and *Y* direction over a 2 × 2 mm area. 

### 2.9. Cell’s Growth Conditions

Osteoblast-like cells were acquired from Bioresource Collection and Research Center (Hsinchu, Taiwan) and were maintained at 37 °C in humidified incubators with 5% CO_2_/95% air in the specific culture media following the protocol from our prior publication [31], as described below. Briefly, MG-63 cells were cultured in Dulbecco’s modified Eagle’s medium (DMEM; HyClone, Logan, UT, USA) and supplemented with l-glutamine (4 mmol/L), 10% fetal bovine serum, and 1% penicillin-streptomycin. The confluent cells allowed to grow until passage 3 using 0.05% trypsin-EDTA. The final concentration was adjusted to 1 × 10^4^ cells/mL and then aliquoted on top of the GDP-treated Zr and Ctrl-Zr discs inside 24-well Petri dishes (Nunclon; Nunc, Roskilde, Denmark).

### 2.10. Cell Vitality Assay

On days 1, 3, and 5, the cell viability was evaluated using a 3-4,5-dimethylthiazol-2-yl)-2,5-diphenyltetrazolium bromide (MTT) kit (Roche Applied Science, Mannheim, Germany). After adding the colorimetric substrate and incubating for 4 h at 37 °C according to the manufacturer’s instructions, alive cells converted the MTT into a formazan dye. The formazan dye with the addition of dimethyl sulfoxide (DMSO) for 10 min changed the color from yellow to dark blue, which was quantified using an ELISA reader (Model 2020, Anthos Labtec Instruments, Eugendorf, Wals, Austria) at a wavelength of 540/570 nm. Three discs were evaluated in each group.

### 2.11. ALP Activity Assay

An ALPase Activity Assay kit (BioVision, Cat # 412-500, Milpitas, CA, USA) can be used for the detection of ALP activity in serum (plasma), tissue, cells, and other samples. ALP is a group of cytomembrane-related enzymes with hydrolysis and transfer activity that acts on a variety of phosphate substrates. Alkaline phosphatase decomposes benzene disodium phosphate to produce free phenol and phosphoric acid. Phenol reacts with 4-aminopyridine in alkaline solution and oxidizes with potassium ferricyanide to form a red quinone derivative. The enzyme activity can be calculated indirectly by measuring the OD value.

### 2.12. Real-Time Polymerase Chain Reaction (qPCR)

The total RNA was extracted using Trizol. Freshly isolated RNA was reverse-transcribed using a high-capacity cDNA reverse transcription kit (Thermo Fisher, Cat#4368814, Waltham, MA, USA) following the manufacturer’s recommendations. The sequences of gene primers used for the real-time PCR were as follows: BSP forward 5′-GCGAAGCAGAAGTGGATGAAA-3′ and reverse 5′-TGCCTCTGTGCTGTTGGTACTG-3′; OC forward 5′-CGCCTGGGTCTCTTCACTAC-3′ and reverse 5′- CTCACACTCCTCGCCCTATT-3′; GAPDH forward 5′-TGCACCACCAACTGCTTAGC-3′ and reverse 5′-GGCATGGACTGTGGTCATGAG-3′. All primers were mixed with SYBR^™^ Green PCR Master Mix (Thermo Fisher, Cat#4344463, Madison, WI, USA) and examined using a LightCycler^®^ 96 Instrument (Roche).

### 2.13. P. gingivalis Cultivation, Adhesion, and Growth

The *P. gingivalis* strain ATCC 33277 (Microbiologics Ltd., Saint Cloud, MI, USA) was grown on TryPtic Soy Agar (ATCC Medium: 260 TryPtic Soy Agar) under an anaerobic condition with 85% N_2_, 5% H_2_, and 10% CO_2_ at 37 °C. After 3 days, the bacteria were inoculated into a peptone yeast extract and incubated under 85% N_2_, 5% H_2_, and 10% CO_2_ at 37 °C for one week. The bacterial concentrations were standardized to an optical density of 0.1, which corresponds to 10^6^ CFU/mL, using a spectrophotometer at 660 nm. To examine the *P. gingivalis* infection on the treated Zr discs, the bacteria were cultivated on the Zr discs at a concentration of 10^6^ CFU/mL and harvested at different times. The *P. gingivalis* suspension was centrifuged at 2000 rpm at 18 °C for 5 min and the resulting bacterial pellet was washed twice. The bacterial suspension was diluted to an optical density of 0.3. Alamar Blue/Resazurin (0. 75 g/ml aqua dest; Sigma–Aldrich) was used to examine the degree of bacterial adhesion. The bacterial growth after adhesion onto discs was examined later. Samples were gathered at 1–5 days after treatment. Each Zr disc was rinsed and fixed for SEM examination. After that, specimens were coated with gold and palladium after the critical point and were dried and mounted on aluminum stubs. The SEM examinations were undertaken at 20.00 kV and analyzed.

### 2.14. Statistical Analysis

All experimental values are demonstrated as the mean ± standard error of at least three recordings. Student’s *t*-test was used for the analysis. A *p*-value < 0.05 signified statistical significance. Data were analyzed using the Statistical Product and Service Solutions software (version 22.0, IBM Corp., Armonk, NY, USA) and Microsoft Excel.

## 3. Results

### 3.1. Control and GDP-Treated Zr Surfaces

**Figure 1:**
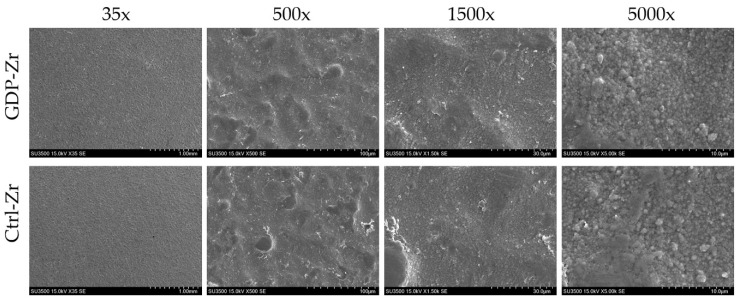
Observation of the Zr discs’ surfaces using SEM.

Zr discs were autoclaved and then divided into two groups, control (Ctrl-Zr) and GDP-treated (GDP-Zr). Scanning electron microscopy (SEM) demonstrated similar irregular surface patterns. Zr discs with a non-plasma treatment had similar surface roughness as the plasma-treated Zr discs (Figure 1).

### 3.2. Similar Atomic Configuration of Zr Discs Using Energy-Dispersive Spectrometry (EDS)

The EDS analysis revealed similar elemental concentrations in the Ctrl-Zr and GDP-Zr discs. After the GDP treatment, the GDP-Zr discs had 64.2 ± 1% Zr, while the Ctrl-Zr discs had 62 ± 1% Zr. Both Zr discs had a similar oxygen weight percentage: 13.6 ± 0.6% for Ctrl-Zr and 12.5 ± 0.6% for GDP-Zr discs.

### 3.3. Implant Surface of Treated Zr Discs Showed Similar Elements in XPS and FTIR Examinations

**Figure 2:**
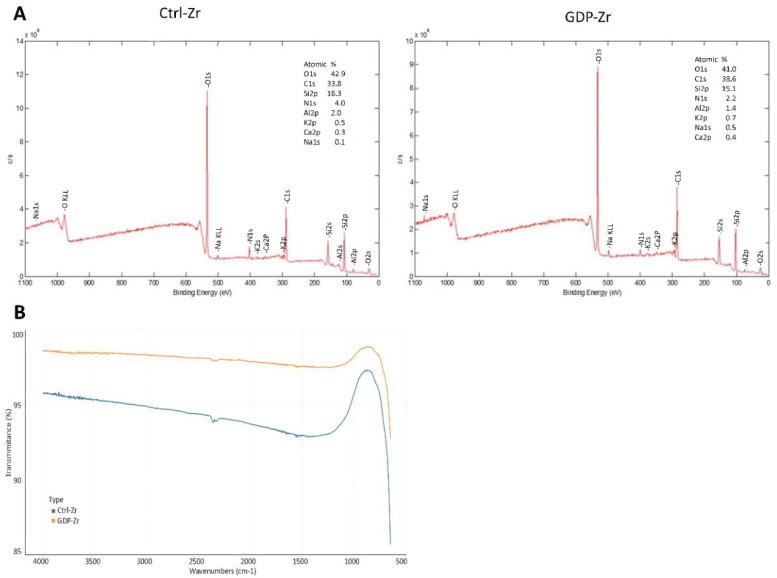
Surface properties were examined using XPS and FTIR analyses. (**A**) The XPS examination showed the surface characteristics of the sample. (**B**) Survey FTIR spectra indicating the atomic peaks of the treated samples. Ctrl-Zr, autoclaved disc; GDP-Zr, glow-discharge-plasma-treated disc.

In order to analyze the elemental changes in the treated Zr discs, XPS and FTIR were carried out (Figure 2A,B). After the GDP treatment, similar results were observed in both tests, indicating that the elements were similarly sustained after treatment.

### 3.4. GDP-Treated Zr Discs’ Surface Roughness and Wettability

**Figure 3:**
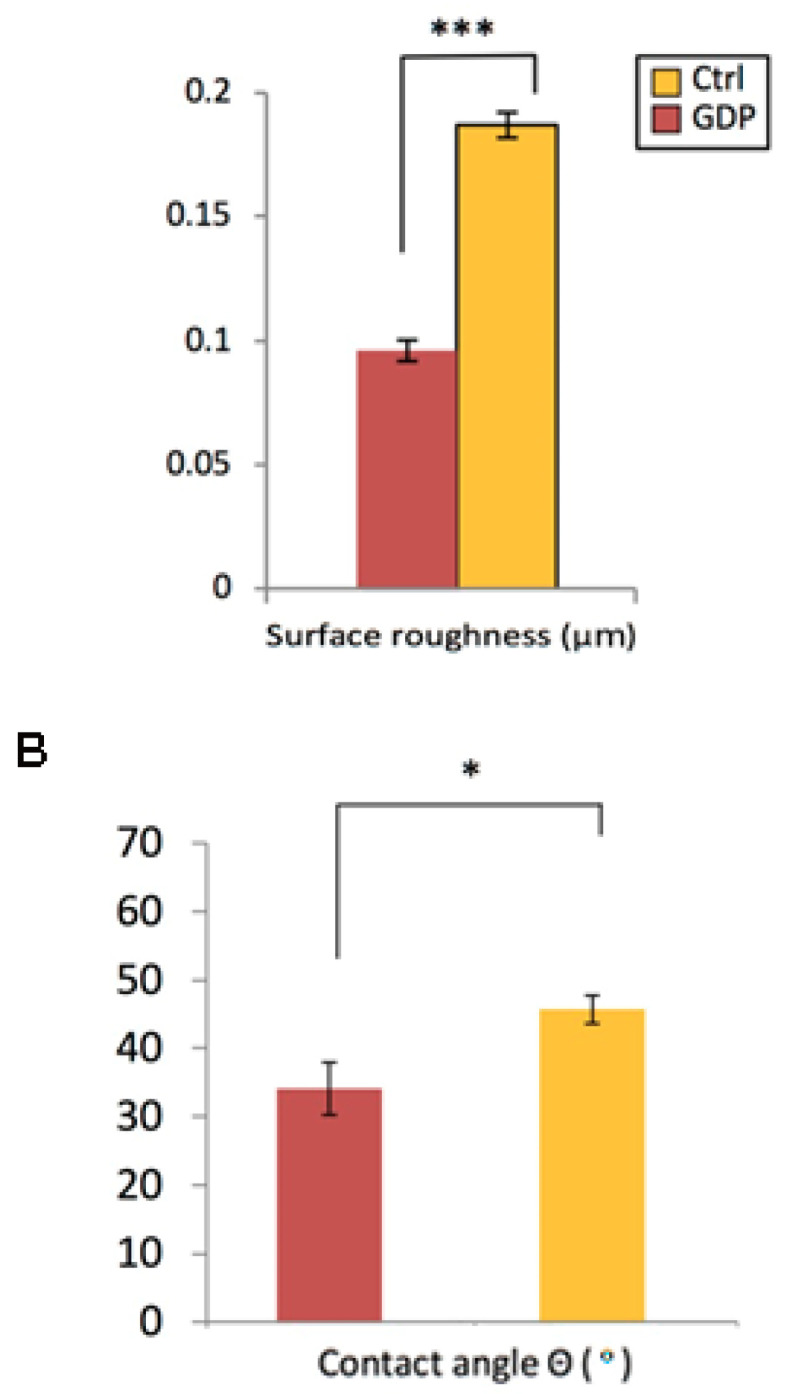
(**A**) The GDP treatment effect on the surface’s roughness (Ra) of Zr discs. The GDP-treated discs showed the lowest roughness, indicating a smoother surface. *** *p* < 0.001. (**B**) The contact angle evaluation for each surface treatment. The index of surface wettability was the contact angle; thus, the formation of the angle by a water droplet refers to the parameter, θ, at the sample surface. Quantitative analysis indicated that the surface wettability of GDP-treated Zr discs was improved compared with the control samples. * *p* < 0.05.

The surface roughness (Ra) values of the treated Zr discs were measured using a profilometer (Figure 3) and the average surface roughness was quantified. The surface roughness values were 0.187 ± 0.005 and 0.096 ± 0.004 μm in the Ctrl-Zr and GDP-Zr groups, respectively (Figure 3), indicating that the GDP-treated Zr discs had a significantly lower roughness than the control discs (*p* < 0.001). A surface wettability test was used to evaluate the surface hydrophilicity of the treated Zr discs, by measuring the contact angle (Figure 3). A flatter water droplet indicates a lower contact angle, suggesting that the disk surface is hydrophilic [40]. We observed relatively lower contact angles in the GDP-treated discs than those in the control discs, showing that the GDP-modified surface displayed better hydrophilicity with a higher surface wettability (*p* < 0.05).

### 3.5. GDP-Treated Zr Discs Exhibited Improved Cell Adhesion

**Figure 4:**
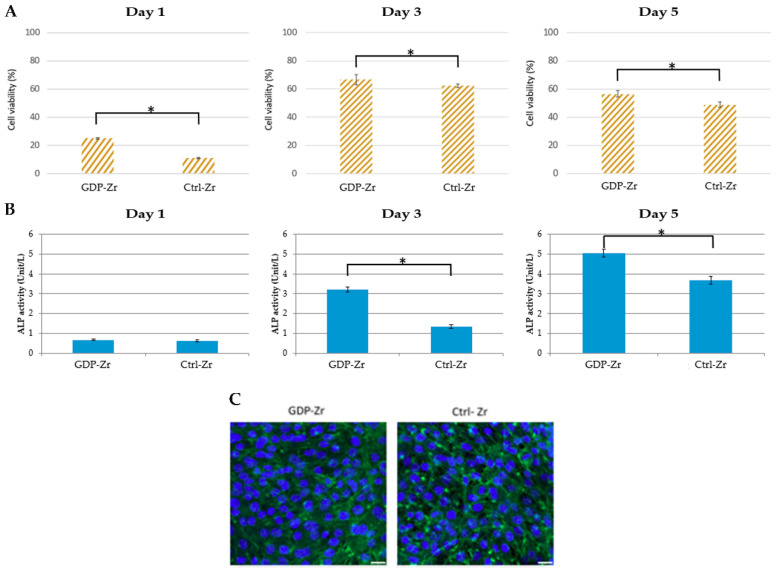
MG-63 cell proliferation on Zr disc surfaces. (**A**) MTT assay of MG-63 cells collected on various days to observe the cell viability on different surfaces. (**B**) Alkaline phosphatase assay (colorimetric) of MG-63 cells collected on various days to observe the alkaline phosphotase (ALP) activity * *p* < 0.05. (**C**) Confocal microscopy observations of MG-63 cells on the treatment surfaces. Images of MG-63 cells on the treated Zr disks and plates at 24 h. Scale bar: 25 μm. GDP-Zr: cells were seeded on GDP-treated Zr disks, Ctrl-Zr: cells on autoclaved Zr disks, Mock: cells on the plate.

Cell viability was assessed using MTT assays of the MG-63 cell line (Figure 4A). From day 1 to day 5 of observation, the MG-63 cells were cultured on top of the GDP-Zr and Ctrl-Zr discs. These findings demonstrated that the GDP-treated Zr disks provided a better environment for cell adhesion than the control discs. Therefore, we conducted confocal microscopy to examine the MG-63 cell attachment. All cells were harvested at 24 h, fixed, and then stained with fluorescent dye to identify nuclei (DAPI, blue) and tubulin (green) (Figure 4C). Confocal microscope findings showed the same tendency with MTT assays of cells having a slightly higher cell density on the GDP-Zr discs than the control Zr discs. Alkaline phosphatase (ALP) activity was also detected (Figure 4B) to determine the relationship with osteointegration. With the increase in the number of culturing days, the ALP activity increased in a time- and dose-dependent manner, indicating that bone formation was induced.

### 3.6. GDP-Treated Discs Induced Osteoblast-Like Cell Osteogenic Markers

**Figure 5:**
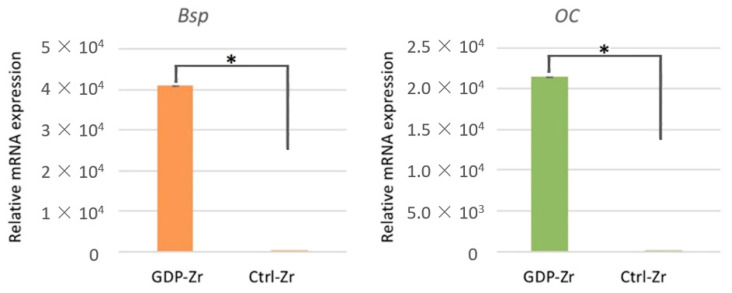
Osteogenic markers in MG-63 cells on day 5. The Bsp and OC relative gene expressions in the MG-63 cells cultivated on plasma-treated Zr discs were higher than the ones cultivated on the control Zr discs. * *p* < 0.05.

To characterize the MG-63 osteoblast phenotype, their gene expressions were analyzed using real-time Polymerase Chain Reaction (qPCR) (Figure 5). Bone sialoprotein (Bsp) and osteocalcin (OC) were targeted for bone formation, and GAPDH expression was investigated to evaluate the use of similar amounts of RNA for RT-PCR. The expression levels of Bsp and OC were observed to be higher in the GDP-treated Zr group on day 5 than the control group. The results of the induction of bone formation in the GDP-treated group are depicted in Figure 5.

### 3.7. GDP-Treated Zr Discs Reduced the P. gingivalis Adhesion and Growth

**Figure 6:**
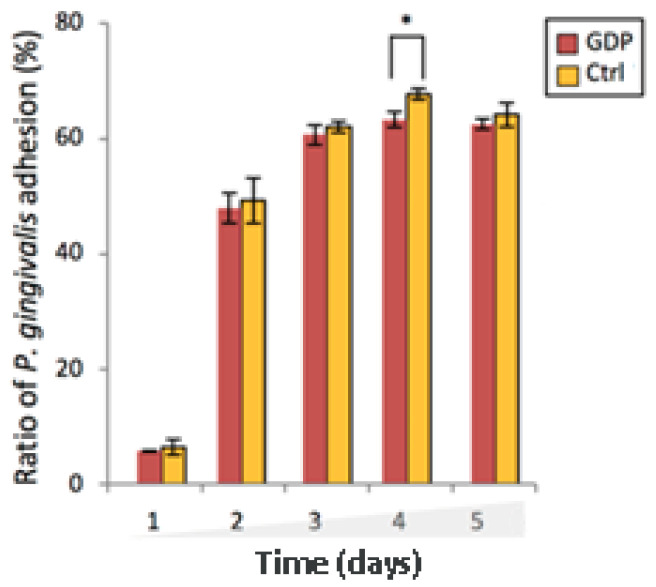
After-treatment adhesion of *P. gingivalis* onto Zr discs. At day 1 post-infection (dpi), the GDP-treated substrate discs demonstrated a lower density of bacterial adhesion. At other time points, the Ctrl-Zr discs exhibited higher bacterial adhesion than the GDP-treated discs. * *p* < 0.05.

The attachment of *P. gingivalis* to Zr-treated discs was quantified at each time point (Figure 6). At all days after the *P. gingivalis* was cultured (dpi), the GDP-treated discs exhibited slightly lower *P. gingivalis* adhesion than the control discs with a statistically significant difference at 4 days (*p* < 0.05).

**Figure 7:**
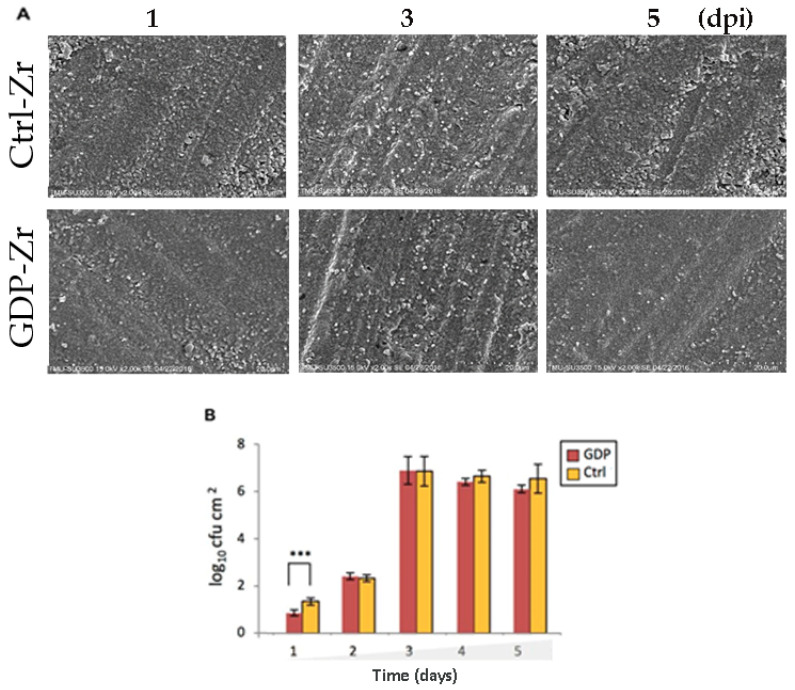
Morphology of the *P. gingivalis* that adhered to the Zr discs after treatment. (**A**) Representative SEM micrographs illustrating the growth of bacteria, showing a relatively lower number of bacteria on the GDP-Zr discs. Magnification: ×2000, scale bar: 20 μm (**B**) The quantified result of the SEM micrographs. *** *p* < 0.001.

The SEM images demonstrated the *P. gingivalis* aggregation (Figure 7A). The colonization of adherent bacteria was similar in the control and GDP groups, only differing by the number of adherent bacteria. The GDP-treated Zr disks demonstrated less bacterial colony formation than the control discs. From 3 dpi onward, the quantified data indicated an increase of *P. gingivalis* accumulation (Figure 7B), implying that the initial bacterial adhesion and growth were related to attachment. Altogether, these findings imply that surface roughness can help the bacterial infection development by benefiting the *P. gingivalis* initial adhesion.

## 4. Discussion

In dentistry, dental implants are widely associated with infections originating from biofilm-producing microbes that trigger inflammation [12,13,14,15,16,17]. Continuous oral inflammation that develops into peri-implantitis is a harmful and intractable problem following dental therapy [15], thereby necessitating the prevention and reduction of microbial biofilm attachment. Some studies have demonstrated that various physical, biological, or chemical treatments of implant metals may reduce *P. gingivalis* adhesion to implant surfaces [7,8,12]. To this end, there has been increasing interest in research focusing on the effective reduction of microbial adhesion and accumulation on implant surfaces. Previous studies have highlighted the indispensable role that implant surface roughness plays in bacterial adhesion [34]. Treatments, such as laser treatment, have gained popularity in dental therapy due to their benefits of low thermal damage; radiofrequency treatment (cold plasma) has also been shown to be capable of inhibiting microbial activity [42,43]. Bacterial colonization occurs during implantation or after osseous integration and interferes with the oral environment and material surface characteristics [44]. GDP treatment has been identified as a method for cleaning and sterilizing metal-based materials, and its use on implant material surfaces, such as Ti, was found to advance cell attachment by modifying the surface roughness and wettability. Meanwhile, other studies investigating the effect of GDP treatment on Zr implants, which are gaining popularity and may replace conventional Ti implants because of their superior esthetic properties, have also reported changes in wettability and osseointegration [6,9]. To better understand the in vitro characteristics of material surfaces involved in the biofilm formation of *P. gingivalis* on Zr implants, disc surfaces were treated with GDP and the interaction between the Zr discs’ surfaces after GDP treatment and *P. gingivalis* infection was investigated. We found that the surface roughness of Zr after GDP treatment positively correlated with *P. gingivalis* proliferation, indicating that GDP-treated Zr discs were slightly less susceptible to bacterial adhesion than control Zr discs (Figure 7). The differences in bacterial adhesion to Zr discs after the GDP treatment were likely due to the bioinert nature of the material, the characteristics of *P. gingivalis* (Figure 6 and Figure 7), and more hydrophilicity after the treatment using argon (Ar-GDP) at 85 W and 13.56 MHz. Roh et al. demonstrated that in vitro 3D PLGA/n-HAp/-TCP composite scaffolds have hydrophobic properties and higher hydrophilicity after being etched with oxygen plasma at 100 W and 13.56 MHz [45]. Despite not using the same element for the GDP treatment, our study employed the same radio frequency and similar power as Roh et al., where both studies showed higher bioactivity on the samples after receiving GDP treatment.

During the initial stages of the *P. gingivalis* adhesion, bacterial attachment to the disc’s surface was benefited due to the higher roughness, indicating that a lower surface roughness resulted in lower bacterial adherence (Figure 6). Thus, since the GDP-treated Zr implant obtained moderately a smoother surface, this could potentially make GDP treatment on Zr implants a clinical approach that periodontists could use to remove bacteria and reduce its adhesion. A GDP surface treatment of Zr implants could effectively be used to reduce peri mucositis and avoid the development of peri-implantitis without damaging the Zr dental implant’s surface. Other studies have also found that GDP-treated Zr implants can induce hydrophilicity and enhance the process of osseointegration [34,39]. In the present study, hydrophilicity increased after the GDP treatment of the Zr discs, which could be correlated with the cells’ better adhesion and proliferation compared with the control Zr discs during the 5 days of testing. During this time, the osteoblastic differentiation was higher on cells cultivated on the GDP-treated Zr (Figure 4). At the same time, the bacteria colonies were just minimally less developed on the GDP-treated Zr discs, probably due to the 85 W and 13.56 MHz low settings at which the GDP treatment was done, somewhat modifying the Zr disc’s roughness (Figure 3).

The preceding experimental results in this study indicated that the cell density was higher on GDP-treated Zr discs than the control Zr discs (Figure 4). These results indicate that the morphology of MG-63 cells on Zr discs was ideal for growth at a specific contact angle after the GDP treatment, providing a low surface roughness. Based on our study result, a contact angle of approximately 35° on the Zr disc, with an average roughness of 0.096 ± 0.004 μm, provided optimal adhesion and proliferation ability of MG-63 cells. A previous study had similar results with a GDP treatment that reduced the contact angle and eliminated residues from the implant surface while maintaining its physicochemical properties [37,38]. The results showed that the GDP treatment improved the implant surface, effectively reduced the bacterial infection, and sustained the cell growth. Some studies have demonstrated similar results with GDP treatment cleaning and modifying Ti implant surfaces by increasing cell adhesion and spread whilst improving the implant surface roughness and wettability [33,34,35,36]. Taken altogether, the findings in this study revealed that a GDP treatment could represent a suitable procedure for acquiring a smoother implant surface to prevent *P. gingivalis* adhesion and proliferation. Furthermore, GDP-treated Zr discs exhibited slightly better resistance against *P. gingivalis* and open the possibility to be used against other bacteria that are also present in peri mucositis and peri-implantitis infections.

## 5. Conclusions

This study has demonstrated that a GDP treatment can reduce bacterial adhesion and proliferation while stimulating cell attachment and osteoblastic differentiation without damaging the Zr disc’s surface. Therefore, GDP could be considered as a Zr dental implant treatment that reduces *P. gingivalis* growth as an approach for peri mucositis treatment and the avoidance of peri-implantitis.
